# Correlation of dosimetric factors with the development of symptomatic radiation pneumonitis in stereotactic body radiotherapy

**DOI:** 10.1186/s13014-020-1479-6

**Published:** 2020-02-13

**Authors:** Jeffrey M. Ryckman, Michael Baine, Joseph Carmicheal, Ferdinand Osayande, Richard Sleightholm, Kaeli Samson, Dandan Zheng, Weining Zhen, Chi Lin, Chi Zhang

**Affiliations:** 1 Department of Radiation Oncology, University of Nebraska Medical Center, 505 S 45th Street, Omaha, NE 68106 USA; 2 College of Medicine, University of Nebraska Medical Center, Omaha, NE USA; 3 Department of Biostatistics, University of Nebraska Medical Center, Omaha, NE USA

**Keywords:** Radiation pneumonitis, Dosimetry, Stereotactic body radiotherapy

## Abstract

**Background:**

The development of radiation pneumonitis (RP) after Stereotactic Body Radiotherapy (SBRT) is known to be associated with many different factors, although historical analyses of RP have commonly utilized heterogeneous fractionation schemes and methods of reporting. This study aims to correlate dosimetric values and their association with the development of Symptomatic RP according to recent reporting standards as recommended by the American Association of Physicists in Medicine.

**Methods:**

We performed a single-institution retrospective review for patients who received SBRT to the lung from 2010 to 2017. Inclusion criteria required near-homogeneous tumoricidal (α/β = 10 Gy) biological effective dose (BED10) of 100–105 Gy (e.g., 50/5, 48/4, 60/8), one or two synchronously treated lesions, and at least 6 months of follow up or documented evidence of pneumonitis. Symptomatic RP was determined clinically by treating radiation oncologists, requiring radiographic evidence and the administration of steroids. Dosimetric parameters and patient factors were recorded. Lung volumes subtracted gross tumor volume(s). Wilcoxon Rank Sums tests were used for nonparametric comparison of dosimetric data between patients with and without RP; *p*-values were Bonferroni adjusted when applicable. Logistic regressions were conducted to predict probabilities of symptomatic RP using univariable models for each radiation dosimetric parameter.

**Results:**

The final cohort included 103 treated lesions in 93 patients, eight of whom developed symptomatic RP (*n* = 8; 8.6%). The use of total mean lung dose (MLD) > 6 Gy alone captured five of the eight patients who developed symptomatic RP, while V20 > 10% captured two patients, both of whom demonstrated a MLD > 6 Gy. The remaining three patients who developed symptomatic RP without exceeding either metric were noted to have imaging evidence of moderate interstitial lung disease, inflammation of the lungs from recent concurrent chemoradiation therapy to the contralateral lung, or unique peri-tumoral inflammatory appearance at baseline before treatment.

**Conclusions:**

This study is the largest dosimetric analysis of symptomatic RP in the literature, of which we are aware, that utilizes near-homogenous tumoricidal BED fractionation schemes. Mean lung dose and V20 are the most consistently reported of the various dosimetric parameters associated with symptomatic RP. MLD should be considered alongside V20 in the treatment planning process.

**Trial registration:**

Retrospectively registered on IRB 398–17-EP.

## Introduction

Stereotactic body radiotherapy (SBRT) is the standard of care for inoperable stage I non-small cell lung cancer (NSCLC), with a local control rate of approximately 95% [1]. Lung SBRT has also demonstrated a progression-free survival benefit in the setting of oligometastatic disease in two recent landmark phase II trials [2, 3]. When considering non-operable stage I NSCLC and the emerging oligometastatic paradigm, the prevalence of lung SBRT in academic centers and throughout the community will continue to increase in the coming years.

Symptomatic radiation pneumonitis (RP) is a well-known subacute side effect of SBRT with reported occurrences ranging from approximately 10–20% of patients treated with commonly used fractionation schemes [4–7]. Symptomatic RP generally occurs within 1 year, typically within 3–6 months, following completion of SBRT, [8–11]. Although radiation-induced lung toxicities (RILTs) are commonly asymptomatic or manageable, some cases are symptomatic with a risk of mortality [12–14]. Historical reporting of symptomatic RP in the context of SBRT has been heterogeneous, further complicating this inherently complex analysis in need of standardized reporting measures. Unified reporting of results moving forward is necessary to provide clarity into treatment-related toxicities in the modern era.

The American Association of Physicists in Medicine (AAPM) Working Group on Biological Effects of SBRT recently recommended new reporting guidelines for papers discussing toxicity from lung SBRT in late 2018 [5]. This work investigates carefully selected patients who were treated with therapeutic doses of SBRT as recommended by Hypofractionated Treatment Effects in the Clinic (HyTEC) [15]. Although the addition of mean lung dose (MLD) to V20 has been suggested as a useful dosimetric constraint [16, 17], no current ongoing clinical trial recommends MLD as a preferred constraint. This work will focus on Vdose and MLD as potential useful constraints according to the new AAPM reporting standards, with the goal of generating logistic regression analysis curves in order to predict the probability of symptomatic radiation pneumonitis.

## Materials and methods

We retrospectively gathered information on all patients treated with SBRT at our institution from 2010 to 2017. Inclusion requirements were patients with at least 6 months of follow up after completion of SBRT (*n* = 91) or documented evidence of symptomatic RP with less than 6 months of follow up (*n* = 2). Near equivalent tumoricidal (α/β = 10 Gy) biological effective dose schemes (BED10) of 100–105 Gy were required in the interest of reporting homogeneous fractionation schemes as recommended by the AAPM (e.g., 50/5, 48/4, 60/8) [5, 15]. Diagnosis of RP required clinical symptoms (i.e. cough or dyspnea requiring increased steroids from baseline with or without interference in activities of daily living) and radiographic evidence, to qualify as an event. As a result, all patients in this study deemed to have “symptomatic RP” were Grade 2+ per CTCAE v3.0/4.0/5.0 or Grade 3+ per RTOG toxicity grading criteria, similarly as analyzed by the AAPM [5]. Chart reviewers recorded patient and tumor characteristics, and were blinded to DVH parameters during the chart review process.

Volumes for all patients were contoured during the original treatment planning process, though a few patients required retrospective contouring of the gross tumor volume (GTV) on free breathing CT. All internal tumor volumes (ITVs) were contoured on 4D CT. Lung volumes subtracted the GTV as recommended by RTOG in a recently published atlas [18]. The difference between ITV and GTV volumes were recorded to investigate the potential impact of tumors with large integrated volumes. Heterogeneity corrections were applied to all patient plans, but with a variety of treatment planning systems and dose algorithms including Eclipse AAA (Varian Medical Systems, Palo Alto, USA) for 58 patients, iPlan PBC (Brainlab AG, Feldkirchen, Germany) for 34 patients, and Pinnacle CCC (Philips Medical Systems, Fitchburg, USA) for 1 patient. For the 8 patients with symptomatic RP, 4 were calculated with Eclipse AAA and 4 with iPlan PBC. Conformity index and gradient index were calculated for each patient according to RTOG 0813, with linear interpretation as required. Velocity 4.0, an image registration and post-processing program, was utilized for tabulating and recording DVH parameters (Velocity, Varian Medical Systems, 2019).

No patients with severe interstitial lung disease (ILD) were included in this study due to institutional preference to not treat these patients to therapeutic doses of SBRT [15], given known increased risk of severe toxicity with baseline severe ILD [11, 19, 20] Severe ILD was defined as advanced cystic changes or disease involving more than 50% of the entire pulmonary volume [19]. For patients with simultaneously treated lesions, dosimetric data from the overall treatment plan was included, though ipsilateral and contralateral lung values could only be calculated if the synchronously treated lesions were located within the same lung.

For patients with multiple encounters, either the encounter that resulted in radiation pneumonitis or their last encounter was used for the analysis. Fisher’s Exact tests and Wilcoxon Rank Sums tests were used to assess differences in demographic and clinical characteristics between patients with and without radiation pneumonitis; *p*-values were Bonferroni adjusted for the various radiation dosage tests to accommodate for multiple testing within each of the following measurement types: percent of the lung, cubic centimeters, and MLD. Logistic regressions were conducted to determine predicted probabilities of radiation pneumonitis associated with percentage of lung exposed using univariable models for each radiation dosage variable. Plots were generated to show the relationships between exposure level and radiation pneumonitis or predicted probabilities of radiation pneumonitis. All analyses were performed using SAS software version 9.4 (SAS Institute Inc., Cary, NC).

## Results

Patient and tumor characteristics for all patients are displayed in Table 1. The overall rate of symptomatic RP was 8.6% (*n* = 8/93), with the median time to develop symptomatic RP of 4.2 months (range 0.9–7.4 months). The majority (*n* = 89) of the patient cohort received 48 Gy in 4 fractions (BED10 = 105 Gy) or 50 Gy in 5 fractions (BED10 = 100 Gy), while only four patients received 60 Gy from five to eight fractions. In the 60 Gy cohort, one patient received 60/5 (BED10 = 132 Gy), two patients received 60/8 (BED10 = 105 Gy) and one patient received 60/6 (BED10 = 120 Gy). Two patients from each of the latter 60 Gy cohorts developed symptomatic RP. Radiation dose categories differed between groups (*p* = 0.004). Groups were similar by pack years, age, treatment year, race, sex, smoking status, ECOG performance status, site, histology, prior radiation to lung, and if two lesions were treated simultaneously.

**Table 1:** Select Patient and Tumor Characteristics

Characteristic	No Radiation Pneumonitis (*n* = 85)	Symptomatic Radiation Pneumonitis (*n* = 8)	*P* value
Age, years			
Median	73.5	72.4	0.56^†^
Range	10.1–89.2	50.4–79.8	
Tumor size, cm^a^			
Median	1.8	3.4	**0.002**^†^
Range	0.6–4.0	1.7–5.3	
T stage^a^			
T1 (%)	73 (94.8%)	2 (33.3%)	**0.001**
T2 (%)	4 (5.2%)	4 (66.7%)	
Radiation Dose			
50/5 (%)	57 (67.1%)	6 (75.0%)	**0.004**
48/4 (%)	26 (30.1%)	0 (0.0%)	
60/5–8 (%)	2 (2.4%)	2 (25.0%)	
Treatment year			
2010	1 (1.2%)	0 (0.0%)	0.86
2011	7 (8.2%)	0 (0.0%)	
2012	9 (10.6%)	0 (0.0%)	
2013	10 (11.8%)	0 (0.0%)	
2014	7 (8.2%)	1 (12.5%)	
2015	19 (22.4%)	2 (25.0%)	
2016	24 (28.2%)	4 (50.0%)	
2017	8 (9.4%)	1 (12.5%)	
Race			
Black (%)	9 (10.6%)	0 (0.0%)	1.000
White (%)	74 (87.1%)	8 (100.0%)	
Other (%)	2 (2.4%)	0 (0.0%)	
Sex			
Female (%)	49 (57.6%)	6 (75.0%)	0.46
Male (%)	36 (42.4%)	2 (25.0%)	
Smoking status (missing = 11)			
Not smoking (%)	23 (30.2%)	4 (66.7%)	1.000
Current smoker (%)	53 (69.7%)	2 (33.3%)	
Pack years			
Median	40	27.5	0.25
Range	0–180.0	0–82.5	
ECOG Performance status			
0 (%)	25 (29.4%)	2 (25.0%)	0.82
1 (%)	43 (50.6%)	4 (50.0%)	
2 (%)	15 (17.7%)	2 (25.0%)	
3 (%)	2 (2.4%)	0 (0.0%)	
Site^a^			
RUL (%)	21 (26.9%)	0	0.06
RML (%)	5 (6.4%)	0	
RLL (%)	23 (29.5%)	2 (33.3%)	
LUL (%)	18 (23.1%)	1 (16.7%)	
LLL (%)	11 (14.1%)	2 (33.3%)	
Liver (%)	0 (0.0%)	1 (16.7%)	
Histology			
Adenocarcinoma (%)	36 (42.4%)	3 (37.5%)	0.87
Squamous cell carcinoma (%)	25 (29.4%)	3 (37.5%)	
No biopsy (%)	9 (10.6%)	1 (12.5%)	
Metastatic non-lung primary (%)	6 (7.1%)	1 (12.5%)	
NOS/other (%)	8 (9.4%)	0 (0.0%)	
SCLC (%)	1 (1.2%)	0 (0.0%)	
Prior Radiation to Lung			
Yes, SBRT (%)	11 (12.9%)	1 (12.5%)	0.27
Yes, CCRT (%)	6 (7.1%)	2 (25.0%)	
Yes, Mantle field (%)	1 (1.2%)	0 (0.0%)	
No (%)	67 (78.8%)	5 (62.5%)	
Two lesions treated simultaneously			
Yes (%)	8 (11.5%)	2 (25.0%)	0.21
No (%)	77 (88.5%)	6 (75.0%)	

Abbreviations: *SBRT* Stereotactic Body Radiation Therapy, *CCRT* Conventionally fractionated concurrent chemoradiation therapy. Symptomatic Radiation Pneumonitis = RTOG G3+ or CTCAE G2+ RP

Bolded *P*-values indicate statistical significance

All *P*-values are from Fishers test unless otherwise noted

^a^Values omit synchronously treated lesions due to multiple values per patient

^†^*P*-values from Wilcoxon Rank Sum tests

**Fig. 1:**
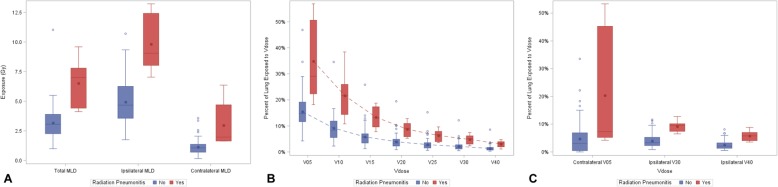
Dosimetric factors and their association with the development of Symptomatic Radiation Pneumonitis. *Abbreviations:* MLD = Mean Lung Dose; V_dose_ is the percent of lung receiving greater than or equal to “dose” (in Gy). Symptomatic Radiation Pneumonitis = RTOG G3+ or CTCAE G2–3+ RP. *Key*: Boxes represent interquartile ranges of dose levels, stars represent mean dose, and circles represent outliers. Differences between asymptomatic patients and patients with symptomatic radiation pneumonitis are statistically significant at each radiation level for **a**) MLD, **b**) Total V_dose_ and **c**) select contralateral and ipsilateral values. The asymptomatic far outlier in panel B represent the same patient (see Table 7 and Individual Patient Data in the Appendix). All V_dose_ values in Panels B and C besides cV5 and iV40 hold statistical significance when evaluated as cubic centimeters (see Table 5, Figs. 3-4 in the Appendix)

Volume, conformity, intermediate dose spillage and MLD characteristics are displayed in Table 2 and Fig. 1. A subset analysis demonstrating continued statistical significance when excluding synchronously treated lesions from conformity index is available in the Table 5 in Appendix. There was no statistically significant association with intermediate-dose spillage and the development of symptomatic RP. Dose Volume Histogram (DVH) characteristics are displayed in Table 3. All of the total lung Vdose metrics, except contralateral V5 (cV5) or ipsilateral V40 (iV40), hold statistical significance when evaluated as cubic centimeters (see Table 6, Figs 3-4 in Appendix). Figure 1 demonstrates a pictorial representation of select data displayed in Tables 2 and 3.

**Table 2:** Volume, Conformity, Intermediate dose spillage and Mean Lung Dose characteristics

Characteristic	No Radiation Pneumonitis (*n* = 85)	Symptomatic Radiation Pneumonitis (*n* = 8)	*P* value
Gross Tumor Volume (GTV), cm^3^			
Median	3.0	15.2	**< 0.001**
Range	0.3–24.3	3.4–41.7	
Integrated Tumor Volume (ITV), cm^3^			
Median	6.0	35.4	**< 0.001**
Range	0.8–39.9	9.0–151.8	
Planning Tumor Volume (PTV), cm^3^			
Median	24.9	77.9	**< 0.001**
Range	5.7–133.3	32.9–370.5	
Prescription dose (RxV), cm^3^			
Median	26.3	78.1	**< 0.001**
Range	6.2–135.1	34.3–361.9	
ITV minus GTV, cm^3^			
Median	3.2	10.2	**0.002**
Range	0–25.8	5.6–33.0	
Conformity Index (RxV / PTV)			
Median	1.05	1.00	**0.04**
Range	0.89–1.44	0.98–1.09	
Intermediate Dose Spillage^a^ (R50V/PTV)			
No deviation (%)	36 (42.4%)	2 (28.6%)	0.78^†^
Minor deviation (%)	45 (52.9%)	5 (71.4%)	
Major deviation (%)	4 (4.7%)	0 (0.0%)	
Total Mean Lung Dose, Gy			
Median	3.1	7.0	**< 0.001**^‡^
Range	1.0–11.0	4.1–9.6	
Contralateral Mean Lung Dose, Gy			
Median	1.1	2.0	**0.001**^‡^
Range	0.2–3.6	1.6–6.4	
Ipsilateral Mean Lung Dose, Gy			
Median	4.7	9.1	**< 0.001**^‡^
Range	1.8–10.7	7.0–13.2	

*RxV* Volume receiving prescription dose, *R50V* Volume receiving 50% of the prescription dose. Symptomatic Radiation Pneumonitis = RTOG G3+ or CTCAE G2+ RP

^a^Of the entire cohort, only one patient was not able to have Intermediate Dose Spillage calculated due to a large PTV size of 370 cm^3^. This patient developed symptomatic radiation pneumonitis

Bolded *P*-values indicate statistical significance

All *P*-values are from Wilcoxon Rank Sum test unless otherwise noted

^†^*P*-value from Fischer exact test

^‡^Bonferroni Adjusted *P*-value

**Table 3:** Dose Volume Histogram Characteristics

Characteristic	No Radiation Pneumonitis (*n* = 85)	Symptomatic Radiation Pneumonitis (*n* = 8)	*P* value
Contralateral Lung V5, %			
Median	3.1%	7.4%	**0.05**
Range	0.0–33.6%	4.2–53.4%	
Ipsilateral Lung V30, %			
Median	3.3%	9.6%	**0.001**
Range	0.8–11.6%	6.5–12.8%	
Ipsilateral Lung V40, %			
Median	2.1%	5.8%	**0.003**
Range	0.5–8.1%	3.6–8.8%	
Total Lung V5, %			
Median	14.7%	29.1%	**0.001**
Range	4.2–46.9%	18.2–56.9%	
Total Lung V10, %			
Median	8.4%	21.7%	**0.001**
Range	2.2–34.6%	10.8–38.4%	
Total Lung V12.4, cm3			
Median	223 cm3	372 cm3	**0.004**
Range	47–789 cm3	311–932 cm3	
Total Lung V13.5, cm3			
Median	195 cm3	333 cm3	**0.004**
Range	41–735 cm3	283–829 cm3	
Total Lung V15, %			
Median	5.4%	13.2%	**0.001**
Range	1.2–25.8%	7.8–18.8%	
Total Lung V20, %			
Median	3.4%	9.1%	**0.001**
Range	0.8–19.4%	5.3–12.8%	
Total Lung V25, %			
Median	2.4%	6.7%	**0.001**
Range	0.6–15.2%	3.6–9.6%	
Total Lung V30, %			
Median	1.8%	5.1%	**0.002**
Range	0.5–12.1%	2.4–7.4%	
Total Lung V40, %			
Median	1.1%	3.3%	**0.003**
Range	0.3–8.6%	1.1–4.7%	

Abbreviations: V_dose_ is the percent of lung receiving greater than the “dose” (in Gy). Symptomatic Radiation Pneumonitis = RTOG G3+ or CTCAE G2+ RP

*P*-values from Wilcoxon Rank Sum tests, with all values Bonferroni adjusted. Bolded *P*-values indicate statistical significance

**Fig. 2:**
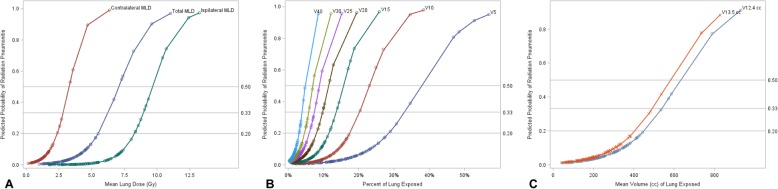
Predicted probability of Symptomatic Radiation Pneumonitis by Radiation Dose. *Abbreviations:* MLD = Mean Lung Dose; V_dose_ is the percent of lung receiving greater than or equal to “dose” (in Gy). Symptomatic Radiation Pneumonitis = RTOG G3+ or CTCAE G2–3+ RP. Plots show predicted probabilities of symptomatic radiation pneumonitis associated with percentage of lung exposed for various dosimetric values, derived from logistic regressions, for **a**) Mean Lung Dose, **b**) Total Lung Values and **c**) Volume of lung receiving 12.4 Gy and 13.5 Gy in cubic centimeters. Probabilities of 20, 33, and 50% are indicated with reference lines. Points on each curve represent individual patient data

**Fig. 3:**
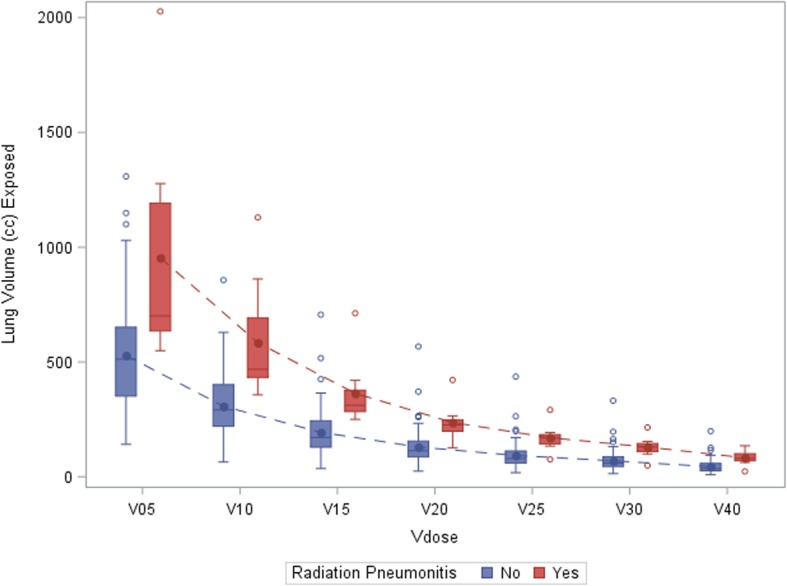
Median Volume Lung Exposure (in cubic centimeters) with Interquartile Ranges by Level. Differences between asymptomatic patients and patients with symptomatic radiation pneumonitis are statistically significant at each radiation level

**Fig. 4:**
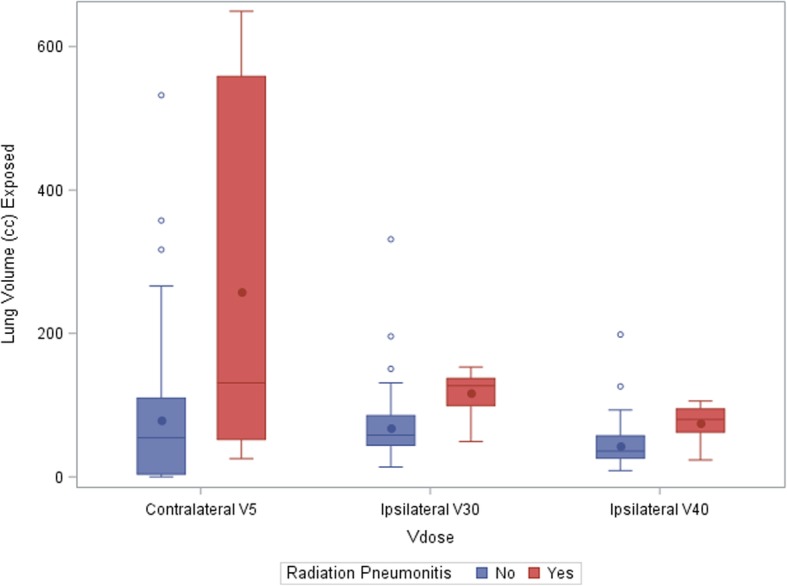
Select Contralateral and Ipsilateral Median Lung Exposure (in cubic centimeters) with Interquartile Ranges by Dose Level. Differences between asymptomatic patients and patients with symptomatic radiation pneumonitis is only statistically significant at the percent of the ipsilateral lung receiving 30 Gy level

Figure 2 displays percentage of lung exposed values with their associated predicted probabilities of developing symptomatic RP, for each dosimetric value; Table 4 displays specific values of percentage of lung exposed which are associated with a predicted probability of symptomatic radiation pneumonitis of 20, 33% or 50% for each dosimetric value. For example, a 33% predicted probability of developing symptomatic RP is associated with a V20 of 9.7% or an MLD of 6.3 Gy, respectively.

**Table 4:** Predicted Probability of Symptomatic Radiation Pneumonitis

Metric	Predicted Probability			C-statistic
	20%	33%	50%	
V5	28.5%	33.0%	37.6%	0.918
V10	17.5%	20.2%	23.0%	0.918
V15	11.6%	13.6%	15.7%	0.935
V20	8.1%	9.7%	11.5%	0.928
V25	6.0%	7.4%	8.8%	0.916
V30	4.7%	5.8%	7.0%	0.904
V40	3.1%	3.9%	4.8%	0.885
V12.4	451.0 cc	538.2 cc	629.2 cc	0.893
V13.5	407.7 cc	491.5 cc	579.1 cc	0.890
Total MLD	5.5 Gy	6.3 Gy	7.1 Gy	0.925
Contralateral MLD	2.4 Gy	2.8 Gy	3.3 Gy	0.929
Ipsilateral MLD	8.3 Gy	9.0 Gy	9.6 Gy	0.952
Contralateral V5	19.3%	25.7%	32.1%	0.821
Ipsilateral V30	8.2%	9.3%	10.4%	0.937
Ipsilateral V40	5.2%	6.1%	6.9%	0.919

Abbreviations: V_dose_ is the percent of lung receiving greater than the “dose” (in Gy). MLD = Mean Lung Dose. Symptomatic Radiation Pneumonitis = RTOG G3+ or CTCAE G2+ RP. For example, a 33% predicted probability of developing symptomatic RP is associated with a V20 of 9.7% or a MLD of 6.3 Gy, respectively

Individual patient data for all patients who developed symptomatic RP is available in Table 7 in Appendix, along with one notable outlier who did not develop symptomatic RP despite concerning DVH parameters. Pertinent images from CT scans, dosimetric characteristics, and potential contributory factors for patients without concerning DVH parameters are also displayed in the Appendix in the Individual Patient Data section. One patient on this study died potentially as a result of SBRT, resulting in an overall rate of death potentially attributable to SBRT of 1.1% (Patient #4). Notably, the use of V20 ≥ 10% alone captured two patients (both with total MLD ≥ 6 Gy, Patients #1–2) while total MLD ≥ 6 Gy alone captured five of the eight patients who developed symptomatic RP (Patients #1–5). No patients with an MLD < 6 Gy exceeded a V20 ≥ 10% on this study. The remaining three patients who developed symptomatic RP were noted to have imaging evidence of moderate interstitial lung disease, inflammation of the lungs from recent concurrent chemoradiation therapy to the contralateral lung, or unique peri-tumoral inflammatory appearance at baseline, suggesting inflammation at baseline was a contributing factor (Patients #6–8).

## Discussion

This work investigates patient characteristics, tumor characteristics, and DVH parameters and their influence in the development of symptomatic RP according to newly recommended reporting requirements by the AAPM [5]. This is the largest report in the literature of which we are aware that solely investigates of tumoricidal near-homogenous BED10 fractionation schemes ranging from 100 to 105 Gy (e.g. 50/5, 48/4, 60/8). Total lung volume exposed to 5–40 Gy (V5 - V40), contralateral lung exposed to 5 Gy, ipsilateral lung exposed to 30 or 40 Gy, contralateral/ipsilateral/total MLD, and volume of GTV, ITV, PTV and ITV minus GTV were significantly higher in those with symptomatic RP than those without (all *p* values < 0.05). There was no significant association between symptomatic radiation pneumonitis and intermediate-dose spillage, age, treatment year, race, gender, smoking status, pack-years, performance status, site, histology, prior radiation to lung, or synchronously treated lesions.

The most commonly recommended constraint for SBRT includes a V20 < 10%, with 15% being an acceptable deviation. Values for V20 ranging from 4 to 12% as the recommended statistically significant endpoint for symptomatic RP have been reported [4, 8, 10, 16, 17, 21, 22], though the majority of studies have reported a V20 less than or equal to 10% to be an appropriate cutoff [5, 9]. This work demonstrated a 33 and 50% predicted probability of developing symptomatic RP to be associated with a V20 of 9.7 and 11.5%, respectively, corroborating well with these recommendations. RTOG 0915 recommends limiting the volume of lung receiving 12.4 Gy (V12.4) < 1000 cc while RTOG 0813 recommends limiting V13.5 < 1000 cc. The median V12.4 was 223 cc (range 47–789 cc) for asymptomatic patients and 372 cc (range 311–932 cc) for patients with symptomatic RP. The median V13.5 was 195 cc (range 41–735 cc) for asymptomatic patients and 333 cc (range 283–829 cc) for patients with symptomatic RP. These values are hypothesis generating, suggesting a lower threshold could be considered for these metrics as a novel planning parameter to optimize treatment-associated patient morbidity further.

Total MLD has been suggested to be an important factor in determining the risk for symptomatic RP. Several studies have reported MLD to be a significant predictor of symptomatic RP, with values ranging from 4 Gy to 14.9 Gy [4, 8, 9, 17, 21, 23, 24]. A recent Meta-analysis suggested 8 Gy as a reasonable cutoff for MLD [5, 7]. Joe Chang’s landmark “No Fly Zone” paper demonstrated both V20 and MLD to be the only dosimetric parameters to be statistically significant on multivariate analysis, with a rate of symptomatic RP 32% for patients with an MLD above 6 Gy [17]. Our results, with a predicted probability of developing symptomatic RP of 33 and 50% for MLD 6.3 Gy and 7.1 Gy, respectively, corroborates well with this data.

Ipsilateral mean lung dose of 10 Gy or higher has been associated with a 26% chance of symptomatic RP (*n* = 7/27) [17]. This work demonstrates a 33% predicted probability of symptomatic RP to be associated with an iMLD ≥9.0 Gy (Table 4, Fig. 2a). Another study which subtracted PTV from lung volumes demonstrated contralateral MLD of 3.6 Gy to be associated with a 37.5% incidence of radiation pneumonitis [25], while this work indicates a 33% predicted probability of symptomatic RP with a cMLD ≥2.8 Gy (Table 4, Fig. 2a). However, contralateral or ipsilateral MLD constraints may not logically apply to synchronously treated lesions in the bilateral lungs. Due to this issue, as well as the lack of studies investigating iMLD and cMLD, it does not seem feasible to make any reasonable conclusions concerning these metrics and their applicability to clinical practice until additional data corroborates with these findings.

Fractionation schemes may have differing rates of pneumonitis, even when BED is nearly equivalent to comparator arms. In this study, 48 Gy in 4 fractions was found to be associated with a 0% occurrence of symptomatic RP (Table 1). This finding was corroborated by another paper [26] which reported that zero patients developed CTCAE G2+ RP with the majority of patients being treated with 48/4 (*n* = 37 of 40 tumors). Another study [22] reported a 13% occurrence of CTCAE G2+ RP with the majority of patients being treated with 48/4 (*n* = 103 of 140 tumors). Further review demonstrated no patients who received 48 Gy in 4 fractions in this study had a MLD above 6 Gy or a V20 higher than 7%. In fact, no patients receiving 48 Gy in 4 fractions in this study exceeded the median values of *any* statistically significant median dosimetric values in Tables 2 and 3, indicating a potential selection preference for 48 Gy in 4 fractions regimen for likely smaller tumors and/or better dosimetric target achievability. Of note, patients treated prior to 2013 did not develop symptomatic RP for reasons similar to the 48 Gy in 4 fraction cohort: All patients treated in this timeframe had T1 tumors, and none had an MLD above 6 Gy or a V20 above 10%.

Only one patient on this study died potentially as a result of SBRT (see Table 7 and Individual Patient Data in the Appendix). Patient #4 had an ultra-central tumor abutting the esophagus and was the only patient to develop RP within 1 month of SBRT. Notably, V20 was 6.0% while exceeding a MLD of 6 Gy at 7.3 Gy. Aside from the elevated mean lung dose, it should be stressed that this patient received 55 Gy to the proximal bronchial tree (PBT) as defined by RTOG 0813, and doses of 50 Gy or higher to the PBT are now contraindicated [27]. This suggests PBT constraints from RTOG 0813 may be inadequate, instead favoring a limit of around 95% of the maximum dose to be preferable (e.g. D0.33cc < 46.5 Gy as suggested by Cleveland Clinic) [28]. Although death caused by central airway injury is rare, the cause of death in this case from other than symptomatic RP is possible. This patient died within 3 months of treatment with severe radiographic RP noted on CT chest at 1 month as compared to pre-treatment baseline.

Conformity index was significantly lower in patients who developed symptomatic RP (*p* = 0.04, Table 2). Of note, three of the four lesions with a conformity index less than 1 were noted to comprise three of the eight overall tumors which were greater than or equal to 3 cm in maximum diameter. To exclude the potential influence of synchronously treated lesions on conformity index, a subset analysis was performed excluding synchronously treated patients (*n* = 10; overall patient number without synchronously treated lesions = 83). Results indicated retained statistical significance (median conformity index for asymptomatic patients of 1.05, range 0.89–1.44; median conformity index for symptomatic RP patients of 0.99, range 0.98–1.03; *p* = 0.013, Table 7in Appendix). This data is hypothesis generating, suggesting more conformal methods such as non-coplanar beams or concentric ring avoidance structures may increase Vdose across a range of dose levels. Newer treatment planning methods such as multicriteria optimization or partial ring avoidance structures may help to lessen the resulting increase in low to intermediate dose bath which may be associated with more conformal treatments.

Synchronously treated lesions were not found to be a risk factor on this study, agreeing with the best available data that synchronous treatments appear to be safe [29]. However, physicians should be wary of synchronously treating lesions without concern for MLD, a reasonable assumption given the recent report of grade 5 pulmonary toxicity in the setting of a low V20 (9.7%) after treatment of one peripheral lesion, one contralateral central lesion, and one liver lesion on SABR-COMET. MLD and potential dose spillage into the lung from the liver lesion were not reported [12]. Similar concerns for the development of symptomatic RP in the setting of a low V20 in the era of immunotherapy have risen at the case report level [30, 31]. Indeed, early data suggests the use of Pembrolizumab within 7 days of SBRT appears to correlate with increased grade 3+ toxicity within the irradiated field [32]. Given G3+ toxicity may contribute towards discontinuation of immunotherapy, additional constraints aside from V20 may be beneficial in the modern era.

Many of the dosimetric values revealed in this work corroborate well with other existing literature on the subject of symptomatic RP, so long as papers with like methodology are compared. Three papers, in particular, are close in range to our total lung mean percent exposure (i.e., total V5-V30) and MLD are Chang 2014 [17], Nakamura [10], and Yamaguchi [22]. Indeed, all three studies recommend *lower* cutoffs than discussed in this study, further driving forth the need to revisit constraints in the modern era. It is likely no coincidence all three studies share many standard features, including accounting for heterogeneity corrections, subtracting GTV from lung volumes, and comparing Grade 0–1 RP to Grade 2+ RP in the setting of near identical fractionation schemes and BED values as were investigated in this report.

Great care must be taken when evaluating studies investigating SBRT and radiation pneumonitis. Many studies do not explicitly mention which treatment volume is excluded from the lung volumes [5, 16, 33], some studies look at only G4+ pneumonitis [11], others group patients into grade 0 versus grade 1–3 RP [34], and still others only look into CTCAE G3+ pneumonitis [19, 20]. It should be noted that steroid administration does not differentiate between CTCAE Grade 2 and Grade 3 toxicity [8]. Wide ranges in MLD have also been demonstrated in probit model parameters when based off fractionation schemes, which are known to be sub-therapeutic according to HyTEC [15], likely resulting in an overestimation of tolerable MLD [5, 21, 23]. As new reporting standards have recently helped to shine a light on these shortcomings [5], we eagerly await future papers on this topic which will provide more clarity on relevant dosimetric endpoints in the modern era.

Limitations of this work include those inherent to retrospective review. Although chart review was blinded to DVH parameters initially, inherent bias exists in patients who were followed up more frequently. Also, excluding patients without at least 6 months of follow up may have resulted in some overestimation, as two patients with documented evidence of symptomatic RP with less than 6 months of follow up were included in this work. Additional limitations include the difficulty in diagnosing symptomatic RP in the setting of patients with underlying lung disease susceptible to community or healthcare-acquired pneumonia, general homogeneity of the studied population, lack of information on quit dates for current smokers versus former smokers, and near-significant heterogeneity of the location of treated lesions between the symptomatic RP and asymptomatic cohorts. It is possible that the incidence of symptomatic RP is underreported for patients who did not receive all care at our institution.

Another limitation is that a variety of dose algorithms were used for the retrospective cohort with varying levels of calculation accuracy especially in terms of heterogeneity corrections. Of them, Pinnacle CCC and Eclipse AAA are known to be more accurate than iPlan PBC, although our previous studies have found that the dose differences among the algorithms are mostly seen for the target especially at the target periphery and much less so for the OARs especially in the low dose regions [35]. Nevertheless, not accounting for the different dose algorithms could introduce additional uncertainty in our results.

## Conclusions

This work has identified many DVH parameters which contribute towards the development of radiation pneumonitis. Future trials should consider incorporation of additional constraints aside from V20, such as MLD, and more stringent values, especially considering the breadth of existing data with similar reporting standards supporting the findings in this work.

## Data Availability

The datasets generated and/or analysed during the current study are not publicly available due to proprietary information from the University of Nebraska Medical Center, but are available from the corresponding author on reasonable request.

## Appendix

**Individual Patient Data from patients who developed Symptomatic Radiation Pneumonitis.**

Correlate with Table 7 in the Appendix.

**Patient #1**: This patient developed symptomatic RP at 3.3 months and received 60 Gy in 8 fractions. Many dosimetric values were above the median when compared to patients who developed symptomatic RP: MLD, iMLD, cMLD, V5-V40, iV30, iV40, and cV5. Notably, V20 was 10.3% while MLD was 9.6 Gy.

**Patient #2**: This patient developed symptomatic RP at 2.3 months and received 50 Gy in 5 fractions to two ipsilateral synchronously treated lesions. The patient had recently completed four cycles of carboplatin and paclitaxel concurrently with 60 Gy in 30 fractions to the contralateral lung 9 months earlier. Irritation from this prior treatment is seen in the contralateral (right) lung prior to treatment as denoted by crosshairs. Many dosimetric values were above the median when compared to patients who developed symptomatic RP: MLD, iV30, and V5-V40. Notably, V20 was 12.3% while MLD was 7.3 Gy.

**Patient #3**: This patient developed symptomatic RP at 7.4 mo and received 50 Gy in 5 fractions to two synchronously treated lesions. Note the sizeable right-sided tumor and bilateral synchronous treatments. This patient died 8 months after treatment with atrial fibrillation with RVR appearing to contribute to demise, 2 weeks after the diagnosis of symptomatic RP. There was only minor evidence of radiation pneumonitis on chest x-ray, therefore we believe it is reasonable to not have attributed this death to radiation pneumonitis. Many dosimetric values were above the median as compared to patients who developed symptomatic RP: MLD, V5, V10, V15, V25, V30. Notably, V20 was only 9.9% while MLD was above 6 Gy at 8.2 Gy.

**Patient #4**: This patient developed symptomatic RP at 0.8 months and received 50 Gy in 5 fractions to a large ultra-central tumor. The gross tumor volume (GTV) was noted to abut the mainstem bronchus and was nearly abutting the esophagus. This patient was the only patient to develop RP within 1 month of SBRT, and died within 3 months of SBRT. The patient presented with worsening cough and shortness of breath and refused admission. Follow up imaging demonstrating fulminant inflammation throughout the ipsilateral lung, therefore we believe it is reasonable this death may be potentially attributable to SBRT. After imaging was obtained demonstrating these findings, the patient was offered admission to the hospital but decided to go home on a steroid burst. Many dosimetric values were above the median as compared to patients who developed symptomatic RP: MLD, cV5, cMLD, iMLD, V5 and V10. Notably, the proximal bronchial tree as defined by RTOG 0813 received 55 Gy while V20 was only 6.0%. MLD was above 6 Gy at 7.4 Gy.

**Patient #5**: This patient developed symptomatic RP at 5.6 months and received 50 Gy in 5 fractions. Note the large liver lesion on the sagittal view (left) with spillage of the PTV into the lung in the setting of inflammation of the posterior lining of the lung best seen in the coronal view (right). Many dosimetric values were above the median as compared to patients who developed symptomatic RP: MLD, iMLD, iV30, iV40, V10, V15, V25, V30, and V40. Notably, V20 was only 9.7% while MLD exceeded 6 Gy at a value of 6.7 Gy.

**Patient #6**: This patient developed symptomatic RP at 5 months and received 60 Gy in 6 fractions. Moderate ILD was present at the time of CT simulation, with moderate ILD defined by the presence of early cystic changes, or disease involving more than one-third of one lung but no more than 50% of the entire pulmonary volume. It is reasonable to suggest underlying inflammatory process at baseline may have contributed towards the development of symptomatic RP. Note the second picture which demonstrates the involvement of the posterior edge of both lungs. Of note, Patient #6 exceeded a few median values of the patients with reported symptomatic RP on this study (i.e., iV30, iV40, V25, V30, V40), while Patients #7–8 did not exceed the median values for any DVH parameters investigated on this study. Notably, MLD was 4.7 Gy while V20 was 8.1%.

**Patient #7**: This patient developed symptomatic RP at 1.2 months and received 50 Gy in 5 fractions. The patient had recently completed concurrent chemoradiation with carboplatin with paclitaxel and 60 Gy in 30 fractions to the contralateral lung just 3 months earlier, at the age of 71. This was the shortest timeframe from CCRT to SBRT of all patients in this study. Note the inflammation present at baseline from prior CCRT in the contralateral lung (see the second image). This patient did not exceed any of the median dosimetric values among patients who developed symptomatic RP (like Patient #8), suggesting inflammation at baseline from prior CCRT may have been a contributing factor. Notably, MLD was only 4.2 Gy while V20 was only 4.7%.

**Patient #8:** This patient developed symptomatic RP at 5.3 months and received 50 Gy in 5 fractions. This was the only tumor on our study which appeared to have a “honeycombing” or cystic appearance and was not biopsied. This patient did not exceed any of the median dosimetric values among patients who developed symptomatic RP (like Patient #7), suggesting inflammation of tumor at baseline was a contributor. Notably, MLD was only 4.1 Gy while V20 was only 5.5%.

**Outlier**: This patient was only 10 years old and had two bilateral lesions treated simultaneously by 50 Gy in 5 fractions. The patient did not develop symptomatic RP despite exceeding median values among patients who developed symptomatic RP. This suggests younger patients may tolerate radiation therapy more readily than older patients, in line with anecdotal evidence. Unfortunately, we could not further tease out this hypothesis given that all other patients in this work were at least 40 years of age or older. It should also be noted the patient also received 12 cycles of gemcitabine and vinorelbine just before irradiation, which might have contributed to a deficient immune system. Notably, MLD was 11 Gy while V20 was 18%. Aside from being the only patient in this work with V20 > 15%, he was also the single patient on our study to have an MLD above 6 Gy who did not develop symptomatic RP.

**Table 5:** Conformity and Intermediate dose spillage for singular and synchronously treated lesions

All patients	No Radiation Pneumonitis (*n* = 85)	Symptomatic Radiation Pneumonitis (*n* = 8)	*P* value
Conformity Index (RxV / PTV)			
Median	1.05	1.00	**0.04**
Range	0.89–1.44	0.98–1.09	
Intermediate Dose Spillage^a^ (R50V /PTV)			
No deviation (%)	36 (42.4%)	2 (28.6%)	0.78^†^
Minor deviation (%)	45 (52.9%)	5 (71.4%)	
Major deviation (%)	4 (4.7%)	0 (0.0%)	
Singular lesions	No Radiation Pneumonitis (*n* = 77)	Symptomatic Radiation Pneumonitis (*n* = 6)	*P* value
Conformity Index (RxV / PTV)			
Median	1.05	0.99	**0.01**
Range	0.89–1.44	0.98–1.03	
Intermediate Dose Spillage^a^ (R50V /PTV)			
No deviation (%)	32 (41.6%)	2 (40.0%)	1.00^†^
Minor deviation (%)	42 (54.5%)	3 (60.0%)	
Major deviation (%)	3 (3.9%)	0 (0.0%)	
Synchronously treated lesions	No Radiation Pneumonitis (*n* = 8)	Symptomatic Radiation Pneumonitis (*n* = 2)	*P* value
Conformity Index (RxV / PTV)			
Median	1.07	1.06	NR
Range	0.96–1.17	1.04–1.09	
Intermediate Dose Spillage (R50V /PTV)			
No deviation (%)	4 (50.0%)	0 (0.0%)	0.56
Minor deviation (%)	3 (37.5%)	2 (100.0%)	
Major deviation (%)	1 (12.5%)	0 (0.0%)	

Abbreviations: *RxV* Volume receiving prescription dose, *PTV* Planned Target Volume, *R50V* Volume receiving 50% of the prescription dose. *NR* Not reportable given small patient number size. Symptomatic Radiation Pneumonitis = RTOG G3+ or CTCAE G2–3+ RP

^a^Of the entire cohort, only one patient was not able to have Intermediate Dose Spillage calculated due to a large PTV size of 370 cm^3^. This patient developed symptomatic radiation pneumonitis

Bolded *P*-values indicate statistical significance

All *P*-values are from Wilcoxon Rank Sum test unless otherwise noted

^†^P-value from Fischer exact test

**Table 6:** Dose Volume Histogram Characteristics (Cubic centimeters of Lung Exposed)

Characteristic	No Radiation Pneumonitis (*n* = 85)	Symptomatic Radiation Pneumonitis (*n* = 8)	*P* value
Contralateral Lung V5, cm3			
Median	54	131	0.30
Range	0–532	25–649	
Ipsilateral Lung V30, cm3			
Median	58	127	**0.02**
Range	14–331	49–153	
Ipsilateral Lung V40, cm3			
Median	36	80	0.06
Range	9–198	23–106	
Total Lung V5, cm3			
Median	513	701	**0.04**
Range	141–1308	549–2027	
Total Lung V10, cm3			
Median	291	468	**0.01**
Range	64–856	357–1129	
Total Lung V12.4, cm3			
Median	223	372	**0.004**
Range	47–789	311–932	
Total Lung V13.5, cm3			
Median	195	333	**0.004**
Range	41–735	283–829	
Total Lung V15, cm3			
Mean	171	311	**0.003**
Range	36–706	250–712	
Total Lung V20, cm3			
Mean	113	226	**0.01**
Range	24–567	125–422	
Total Lung V25, cm3			
Mean	79	169	**0.01**
Range	18–436	75–291	
Total Lung V30, cm3			
Mean	59	130	**0.01**
Range	13–332	49–215	
Total Lung V40, cm3			
Mean	36	81	**0.02**
Range	9–199	23–135	

Abbreviations: V_dose_ is the percent of lung receiving greater than the “dose” (in Gy). Symptomatic Radiation Pneumonitis = RTOG G3+ or CTCAE G2–3+ RP

*P*-values from Wilcoxon Rank Sum tests, with all values Bonferroni adjusted

Bolded *P*-values indicate statistical significance

**Table 7:** Details on patients who developed Symptomatic Radiation Pneumonitis and the Outlier with extreme DVH values who did not develop radiation pneumonitis

	Age	Dose scheme	Synchronously treated lesions?	Largest tumor size	Date from SBRT to RP	Date from SBRT to death	Prior RT to lung? (months)	MLD	V20
Developed Symptomatic Radiation Pneumonitis									
Patient 1	75	60/8	No	3.3 cm	3.3 mo	Alive	SBRT (20 mo)	9.6 Gy	10.3%
Patient 2	50	50/5	Yes	1.6 cm	2.3 mo	Alive	CCRT (9 mo)	7.3 Gy	12.5%
Patient 3	80	50/5	Yes	3.6 cm	7.4 mo	8 mo	No	8.2 Gy	9.9%
Patient 4	68	50/5	No	4.0 cm	0.8 mo	2.7 mo	No	7.4 Gy	6.0%
Patient 5	60	50/5	No	Liver	5.6 mo	Alive	No	6.7 Gy	9.7%
Patient 6	80	60/6	No	2.8 cm	5 mo	Alive	No	4.7 Gy	8.1%
Patient 7	71	50/5	No	1.7 cm	1.2 mo	Alive	CCRT (3 mo)	4.2 Gy	4.7%
Patient 8	74	50/5	No	3.5 cm	5.3 mo	Alive	No	4.1 Gy	5.5%
Outlier who did not develop Symptomatic Radiation Pneumonitis									
Outlier 1	10	50/5	Yes	1.9 cm	n/a	Alive	No	11.0 Gy	18.4%

Only one patient appeared to have experienced death which may have been attributable to symptomatic RP (Patient #4)

This table corresponds to Appendix: Individual Patient Data in the Figures file
