# Correction to: Correlation of dosimetric factors with the development of symptomatic radiation pneumonitis in stereotactic body radiotherapy

**DOI:** 10.1186/s13014-021-01797-3

**Published:** 2021-04-07

**Authors:** Jeffrey M. Ryckman, Michael Baine, Joseph Carmicheal, Ferdinand Osayande, Richard Sleightholm, Kaeli Samson, Dandan Zheng, Weining Zhen, Chi Lin, Chi Zhang

**Affiliations:** 1 Department of Radiation Oncology, University of Nebraska Medical Center, 505 S 45th Street, Omaha, NE 68106 USA; 2 College of Medicine, University of Nebraska Medical Center, Omaha, NE USA; 3 Department of Biostatistics, University of Nebraska Medical Center, Omaha, NE USA

## Correction to: Radiat Oncol (2020) 15:33 10.1186/s13014-020-1479-6

Following publication of the original article [1], we have been notified by the authors about the following corrections in the article.

The authors discovered errata concerning the definition of the V13.5 and V12.4 dose “limits” to the lung. Their work defined these volumes as the minimum volume of lung exposed to the corresponding doses instead of minimum volume of lung spared (MVLS) (Ritter, Matuszak et al. 2017). The section which reads incorrectly is in the Discussion section as follows:

“RTOG 0915 recommends limiting the volume of lung receiving 12.4 Gy (V12.4) < 1000 cc while RTOG 0813 recommends limiting V13.5 < 1000 cc. The median V12.4 was 223 cc (range 47–789 cc) for asymptomatic patients and 372 cc (range 311–932 cc) for patients with symptomatic RP. The median V13.5 was 195 cc (range 41–735 cc) for asymptomatic patients and 333 cc (range 283–829 cc) for patients with symptomatic RP. These values are hypothesis generating, suggesting a lower threshold could be considered for these metrics as a novel planning parameter to optimize treatment-associated patient morbidity further.”

We have performed additional analysis of the MVLS from 12.4 Gy and MVLS from 13.5 Gy, switching these values for the previously reported values of V12.4 and V13.5 in Table 3 (see below). This section should read as:

**Table 3:** Dose volume histogram characteristics

Characteristic	No radiation pneumonitis (n = 85)	Symptomatic radiation pneumonitis (n = 8)	*p* value
Contralateral lung V5, %			0.05
Median	3.1%	7.4%	
Range	0.0–33.6%	4.2–53.4%	
Ipsilateral lung V30, %			0.001
Median	3.3%	9.6%	
Range	0.8–11.6%	6.5–12.8%	
Ipsilateral lung V40, %			0.003
Median	2.1%	5.8%	
Range	0.5–8.1%	3.6–8.8%	
Total lung V5, %			0.001
Median	14.7%	29.1%	
Range	4.2–46.9%	18.2–56.9%	
Total lung V10, %			0.001
Median	8.4%	21.7%	
Range	2.2–34.6%	10.8–38.4%	
MVLS from 12.4 Gy, cm^3^			0.31
Median	3172 cm^3^	2132 cm^3^	
Range	969–6807 cm^3^	1439–3592 cm^3^	
MVLS from 13.5 Gy, cm^3^			0.31
Median	3196 cm^3^	2157 cm^3^	
Range	980–6829 cm^3^	1452–3603 cm^3^	
Total lung V15, %			0.001
Median	5.4%	13.2%	
Range	1.2–25.8%	7.8–18.8%	
Total lung V20, %			0.001
Median	3.4%	9.1%	
Range	0.8–19.4%	5.3–12.8%	
Total lung V25, %			0.001
Median	2.4%	6.7%	
Range	0.6–15.2%	3.6–9.6%	
Total lung V30, %			0.002
Median	1.8%	5.1%	
Range	0.5–12.1%	2.4–7.4%	
Total lung V40, %			0.003
Median	1.1%	3.3%	
Range	0.3–8.6%	1.1–4.7%	

Abbreviations: Vdose is the percent of lung receiving greater than the “dose” (in Gy). Symptomatic Radiation Pneumonitis = RTOG G3 + or CTCAE G2 + RP. MVLS = Minimum Volume of Lung Spared

*p* values from Wilcoxon Rank Sum tests, with all values Bonferroni adjusted. Bolded *p* values indicate statistical significance

“RTOG 0915 recommends the minimum volume of lung spared (MVLS) from 12.4 Gy to be at least 1000 cc to minimize G3+ pneumonitis while RTOG 0813 recommends MVLS from 13.5 Gy to be at least 1000 cc to minimize G3+ pneumonitis. RTOG 0813 also recommends MVLS from 12.5 Gy to be at least 1500 cc to preserve basic lung function. The median MVLS from V12.4 was 3172 cc (range 968–6807 cc) for asymptomatic patients and 2131 cc (range 1438–3592 cc) for patients with symptomatic RP. The median MVLS from V13.5 was 3196 cc (range 980–6829 cc) for asymptomatic patients and 2157 cc (range 1452–3602 cc) for patients with symptomatic RP. MVLS for each parameter were the only two dosimetric parameters in Table 3 which did not reach statistical significance for symptomatic RP (*p* = 0.31). These findings are hypothesis generating, suggesting MVLS from 12.4 Gy and 13.5 Gy may not be as robust of parameters as previously believed.”

The authors apologize for this error and are thankful the results did not change the significance of this work, as MVLS was statistically insignificant in regards to the development of symptomatic radiation pneumonitis.

Bibliography

- Ritter, T. A., et al. (2017). "Application of critical volume-dose constraints for stereotactic body radiation therapy in NRG radiation therapy trials." International Journal of Radiation Oncology Biology Physics 98(1): 34–36.

## References

[1] Ryckman JM (2020). Correlation of dosimetric factors with the development of symptomatic radiation pneumonitis in stereotactic body radiotherapy. RadiatOncol.

